# Cation folding and the thermal stability limit of the ionic liquid [BMIM^+^][BF_4_^−^] under total vacuum

**DOI:** 10.1039/d1ra00741f

**Published:** 2021-04-06

**Authors:** J. Alberto Arroyo-Valdez, Gonzalo Viramontes-Gamboa, Roberto Guerra-Gonzalez, Mariana Ramos-Estrada, Enrique Lima, José L. Rivera

**Affiliations:** Facultad de Ciencias Físico-Matemáticas, Universidad Michoacana de San Nicolás de Hidalgo Morelia Michoacán 58000 Mexico jlrivera@umich.mx; Facultad de Ingeniería Química, Universidad Michoacana de San Nicolás de Hidalgo Morelia Michoacán 58000 Mexico; Laboratorio de Fisicoquímica y Reactividad de Superficies (LaFReS), Instituto de Investigaciones en Materiales, Universidad Nacional Autónoma de México Circuito Exterior S/N, CU, Del. Coyoacán Ciudad de México Mexico

## Abstract

Molecular dynamics simulations reveal the behavior of the bimodal distribution of cation conformations (folded/unfolded) in ionic liquids based on alkylated imidazoles, such as [BMIM^+^][BF_4_^−^]. The alkyl chains of the cations can fold and block interactions between the cations and anions, thereby reducing the cohesivity of the liquid. At room temperature, the folded conformations represent less than one-third of the total conformations. In contrast to the behavior observed during the thermal denaturation of proteins, in ionic liquids, the concentration of folded cations grows when the temperature increases. At the equimolar concentration, the system reaches the reported experimental temperature of thermal stability (similar to the thermal denaturation behavior). There is an outermost layer of cations at the interface that can tilt toward the interface and cover a layer of anions adsorbed at the interface. This interfacial conformation makes the system stable in transverse directions and unstable in the normal direction at temperatures in the region of thermal instability, limiting the rate of vaporization of neutral ion pairs, which are observed as rare events at temperatures as low as 773.15 K.

## Abstract 2

Snapshot of a vaporized neutral ion pair near the liquid layer of [BMIM^+^][BF_4_^−^] under vacuum–liquid equilibrium at 773.15 K.

## Graphical abstract

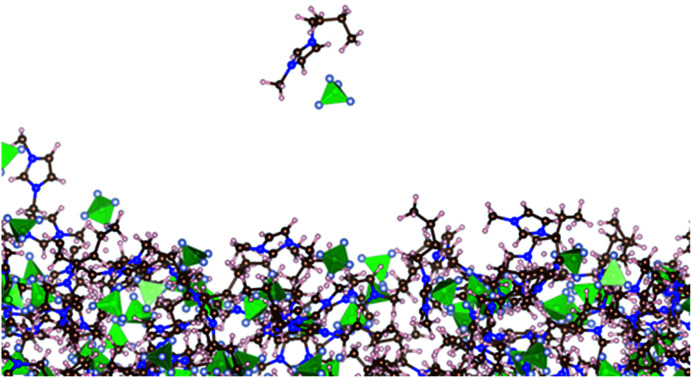

## Introduction

The two-state folded/unfolded model for proteins is a very useful representation for the thermal denaturation of small proteins,^1,2^ individual protein molecules,^3,4^ and proteins with intermediate states that are too unstable to be detected.^5,6^ In this model, each state is favored at different sets of temperatures. At lower temperatures, an enthalpy-driven process favors folded states and at higher temperatures, an entropy-driven process favors unfolded states, with a phase-transition temperature (*i.e.*, melting temperature) characterized by the system's configuration of a 50% concentration of folded (and 50% unfolded) proteins.^7–9^ At this transition temperature, other thermodynamic properties show interesting behaviors; the heat capacity shows a maximum, and after full denaturation of the proteins, the heat capacity increases, a phenomenon commonly associated with hydrophobic domains interacting with the water solvent. The volume of the protein shows two regimes that can be associated with the two-state model and are separated by the transition temperature.^10–12^

Unfolding behavior is not unique to proteins; flexible homopolymer chains with sufficiently short-range interactions in the absence of any solvent also exhibit the folded/unfolded behavior^13^ associated with low-entropy and high entropy states separated by a phase transition temperature (unfolding temperature).^14^ In another study, this temperature was found to be close to the maximum in the heat capacity.^15^ Hydrophobic polymers at diluted conditions near a hydrophilic wall also show a differentiated behavior, fully folding in the bulk of the liquid, folding to 2D structures when adsorbed at the hydrophilic wall and unfolding when excluded from the liquid/vapor interface.^16^

Ionic liquids composed of cations with small alkyl chains, such as those present in imidazole-based ionic liquids, can bend and fold to “scorpion-like” structures due to the relatively strong interaction between the end group of the alkyl chains and the aromatic imidazole.^17^ It is expected that the folding and unfolding of cations in ionic liquids is temperature-dependent. There is a feasible scenario where the foldable large cation and small anion pair behaves like the protein (polymer) and solvent system, and a transition temperature separates the folding and unfolding regimes. Ionic liquids are stable up to the temperatures where the liquid is thermally stable, and then the liquid vaporizes. The value of this temperature has not been as well defined as many of the other thermophysical properties of ionic liquids; for the ionic liquid [BMIM^+^][BF_4_^−^], the reported values are between 588 and 676 K, corresponding to the onset temperature obtained using experimental thermogravimetric analysis.^18–23^ The variance in the reported values could result from impurities.^24^

In the region of thermal stability, ionic liquids not only physically vaporize but also chemically decompose. The ion structures break and recombine as the temperature increases, but this phenomenon is likely unimportant because experiments have shown that, at 413.15 K, only 1 in 340 [BMIM^+^] cations dissociates into imidazole ions while the eliminated *n*-butyl chains join other [BMIM^+^] ions through retro-alkylation processes.^25^

The physical vaporization of ionic liquids is not easy to study because these liquids have extremely low vapor pressure (negligible at room temperature), and they physically vaporize (in conjunction with thermal decomposition) at temperatures hundreds of kelvins over the room temperature.^20^ Recent estimates reported values for the vapor pressure similar to those found in high vacuum conditions, between 10^−7^ and 10^−5^ bar, for [BMIM^+^][BF_4_^−^] at temperatures in the range of 448.15–523.15 K.^26^

The composition of the vapor phase and the gas phase at temperatures over the thermal limit of stability is a topic of ongoing study. Thermal desorption–ionization experiments have shown the possibility of single ions forming the gas phase in induced vaporization at atmospheric pressure.^27^ However, several studies on thin films under ultravacuum conditions in different ionic liquids have favored the formation of aggregates of at least a pair of ions.^28–31^ Leal *et al.*^28^ have studied several ionic liquids at temperatures in the limit of thermal stability (420–650 K) and high vacuum reduced pressures (10^−6^ to 10^−4^ Pa), and found that the gas phase consists of neutral aggregates. Armstrong *et al.*^29^ studied several imidazole-based ionic liquids and also found neutral ion pairs using mass spectroscopy in vaporizations at ultra-high vacuum conditions, and the heat of vaporization depended on the strength of the coulombic interactions. Ballone *et al.*^31^ studied the cohesive energy in neutral and charged clusters of imidazole-based ionic liquids using molecular dynamics simulations and found that neutral clusters are more cohesive than charged clusters. Using free energy calculations, they found that the most expected cluster contains one ion pair at temperatures beyond the limit of thermal stability, but there is also the possibility of clusters with two ion pairs and single ions.

Vaporization can be enhanced, and ionic liquids can be distilled at a measurable rate by placing the equilibrium interface under rotatory movement and ultravacuum conditions.^32^ Earle *et al.* distilled a series of imidazole-based ionic liquids with increasing chain length under rotatory movement at a temperature of 573.15 K, and found that the distillation rate decreases with the length of the chain. Distilled ionic liquids did not chemically decompose into subcomponents of their original structure, which confirms the lack of influence of chemical decomposition near the limit of thermal stability. At room temperature, vapor pressure for the distillation is negligible.

This work was designed to determine the effect of the conformation of the cations on the thermal stability of the system and the interfacial forces that pairs of ions need to overcome to be vaporized.

## Computational details

**Fig. 1:**
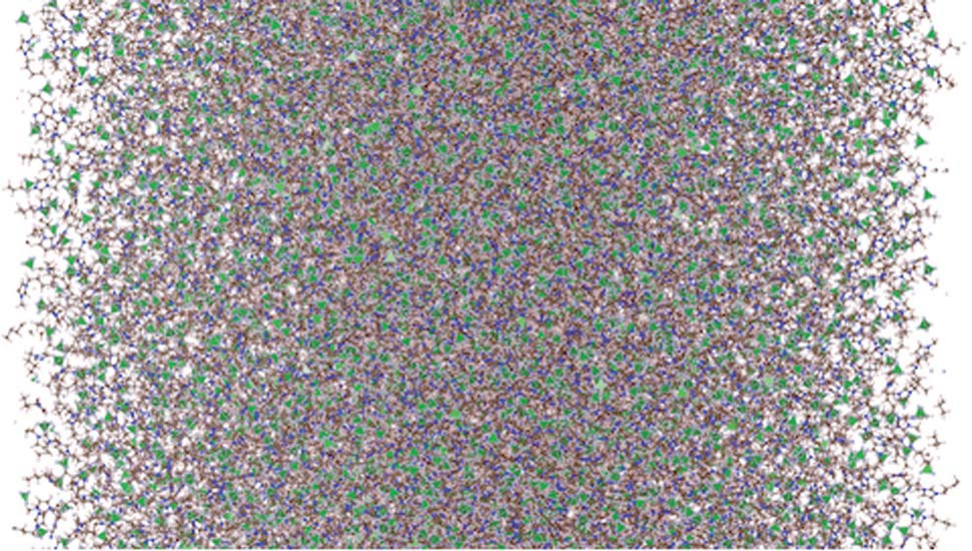
Snapshot of the liquid layer of [BMIM^+^][BF_4_^−^] under vacuum–liquid equilibrium at 298.15 K. The interfaces are located at the horizontal limits of the system. The brown, pink, blue, and gray circles represent carbon, hydrogen, nitrogen, and fluorine atoms. The green tetrahedrals represent boron atoms.^60^

We carried out molecular dynamics simulations of a liquid layer of a [BMIM^+^][BF_4_^−^] ionic liquid surrounded by vacuum, from room temperature, 298.15 K, to 773.15 K. The liquid layer contained two interfaces, under periodic boundary conditions, that mimicked the behavior of infinite-area vacuum/liquid interfaces (Fig. 1). The simulation cell consisted of a parallelepiped with the dimensions 10 × 10 × 48 nm. The interfacial area of the central simulation cell was 100 nm^2^. The system consisted of 4000 pairs (120 000 atoms) of ions of [BMIM^+^][BF_4_^−^], which were simulated using fully flexible atomistic models that included point charges and Lennard-Jones interactions with damped functions in order to accurately describe the interfacial forces.

The intramolecular interactions were calculated using harmonic potentials to account for bond, angle and improper angle vibrations. Dihedral vibrations were accounted using the CHARMM-style expression.^33^ Intermolecular interactions were calculated using the expression:1
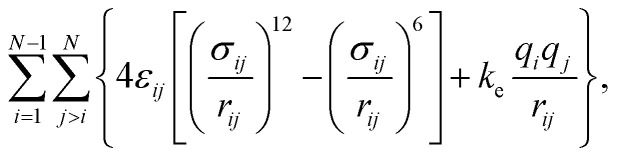
where *N* is the number of interaction sites in the system, *σ*_LJ_ and *ε*_LJ_ are the Lennard-Jones parameters, *r*_*ij*_ is the separation between sites *i* and *j*, *q*_*i*_ is the point charge of site *i*, and *k*_e_ is the Coulomb's constant. The Lennard-Jones interactions were damped using a switching function to mimic the full Lennard-Jones interactions at the interface, and avoid the need of long-ranged corrections to the surface tension, which can reproduce the surface tension of the system but do not properly reproduce the true interfacial dynamics of the system.^34^ The inner/outer cutoff radii employed in this work for the damped Lennard-Jones interactions were 16.5 and 18.15 Å, respectively, which are long enough to account for all the interactions separated by a minimum of 4.5 *σ*_LJ_ (smaller cutoff radii do not mimic the full Lennard-Jones potential). Electrostatic interactions were computed using the particle–particle particle-mesh algorithm^35^ using the outer cutoff radius of the damped Lennard-Jones potential. Intramolecular contributions between atoms separated up to two bonds due to Lennard-Jones and electrostatic interactions were neglected, electrostatic interactions separated by three bonds were scaled by 40%, while Lennard-Jones interactions were not scaled, and interactions for sites separated by four or more bonds were fully accounted. The parameters for [BF_4_^−^] were taken from the work of Wu *et al.*,^36^ while the parameters for [BMIM^+^] were taken from the work of Cadena and Maginn.^37^

The system was simulated using an NVT ensemble (*i.e.*, number of atoms, volume, and temperature constant) and a Nosé thermostat,^38^ using the open-source large-scale atomic/molecular massively parallel simulator (LAMMPS)^39^ with a timestep of 1 fs. The periods of equilibration and property statistics were 1 and 10 ns, respectively. Profiles of density, pressure, and radius of gyration (*R*_g_) were calculated in slabs of 0.1 Å in the normal direction to the interface. The pressure profiles were obtained by calculating the pressure tensors in each slab, using Harasima pressure profiles^40,41^ implemented in LAMMPS.^42^ The profiles were calculated for every 100 timesteps (10^5^ profiles), and the profiles were averaged, correcting the profiles through the center of the layer, which was calculated as the midpoint between the Gibbs dividing surfaces, which were obtained fitting the total density profiles to an expression dependent on the separation to the interfaces in the normal direction, *z*:^43,44^2
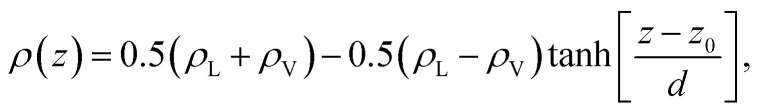
where *ρ*_L_ and *ρ*_V_ are the average liquid and vapor bulk densities, respectively. *z*_0_ is the position of the Gibbs' dividing surfaces, an *d* is a parameter related to the thicknesses of the interfaces.

## Results and discussion

### Density profiles

**Fig. 2:**
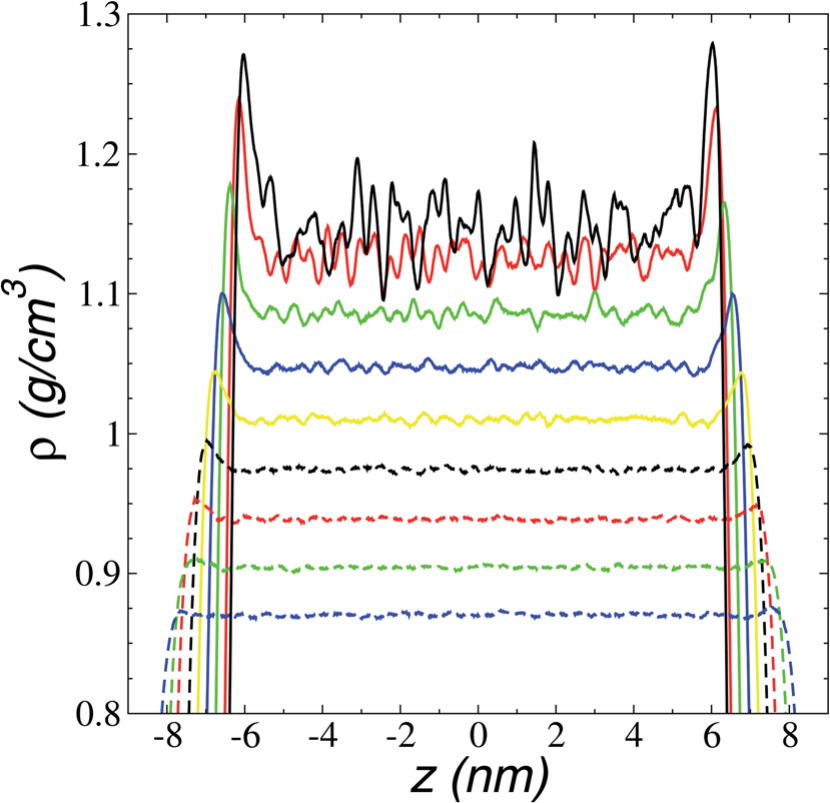
Averaged total density profiles as a function of position in the normal direction to the vacuum–liquid interface for the ionic liquid [BMIM^+^][BF_4_^−^]. Continuous lines represent temperatures of 298.15 K (black), 323.15 K (red), 373.15 K (green), 423.15 K (blue), 473.15 K (yellow), and discontinuous lines 523.15 K (black), 573.15 K (red), 623.15 K (green) and 673.15 K (blue).

We first studied how the components of the ionic liquid are distributed and oriented at the interface through the analysis of the total, ionic, and atomic density profiles. Density profiles of the liquid and vacuum equilibrium averaged for 10 ns are plotted in Fig. 2 for the ionic liquid [BMIM^+^][BF_4_^−^], at temperatures in the range of 298.15–673.15 K. The profiles at temperatures up to 323.15 K show remarkable fluctuations in the central part of the profile, corresponding to the bulk liquid. Those fluctuations can be associated with a solid-like fluid behavior, commonly seen in regular solvents at temperatures near the triple point.^45^ The profiles showed large adsorption peaks at the interfaces, which decreased in magnitude as the temperature of the system increased, and these peaks almost disappear at temperatures in the region of thermal stability. Bulk liquid densities were obtained, averaging the values of the total density profiles in the region between −5 and 5 nm. Bulk liquid densities obtained in the simulation underestimate the only set of experimental values reported in the literature (292.89–391.28 K)^46^ by up to 4.3%, but no other experimental values were found in the literature for comparison.

**Fig. 3:**
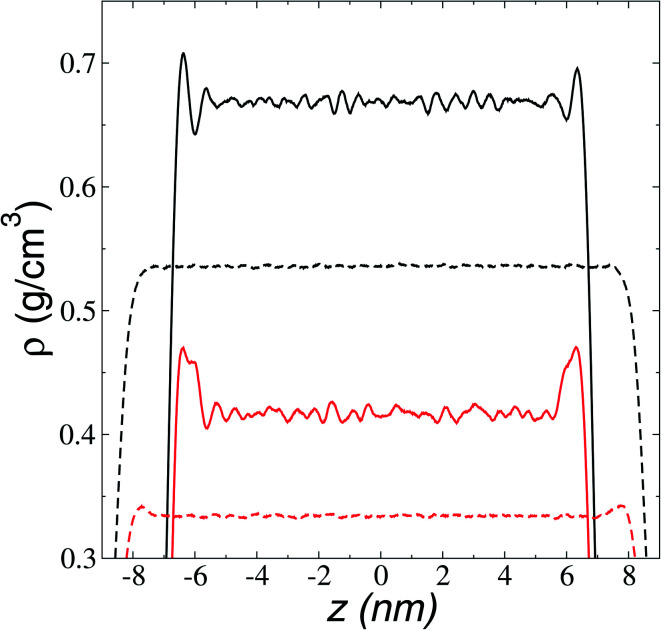
Ionic density profiles of [BMIM^+^][BF_4_^−^] as a function of position in the normal direction to the vacuum–liquid interface. The continuous and discontinuous lines represent profiles at temperatures of 373.15 and 673.15 K, respectively. Black and red lines represent profiles for [BMIM^+^][BF_4_^−^], respectively.

The adsorption peaks at the interfaces are due to an accumulation of one of the components in the binary mixtures, which in ionic liquids corresponds mainly to anions accumulating at the interfaces. In a previous study, we showed that triflate anions accumulated in a zone before the outermost part of the interfaces of the ionic liquid [BMIM^+^][Triflate^−^], with the outermost layer formed by the BMIM cations with alkyl chains pointing out of the interface.^47^ The ionic density profiles for the ionic liquid [BMIM^+^][BF_4_^−^] (Fig. 3) show that the component that accumulated at the liquid–vacuum interface is also the anion group. The anion profiles show large adsorption peaks at room temperature as previously reported, decreasing in magnitude as the temperature is increased. At temperatures near room temperature, the cations also show peaks of adsorption at the interface, which disappear at 473.15 K. The temperature at which the magnitude of the adsorption peaks of anions almost disappears is between 673.15 and 773.15 K and corresponds to the thermal limit of stability.^18–23^ Therefore, the disappearance of the anion peaks can be used as a criterion to determine this temperature limit through molecular simulations or experimental methodologies. The observed accumulation of anions at the interface in molecular dynamics simulations has been used to explain the enhanced adsorption of polar gases by ionic liquids.^48^

### Orientation of the chains at the interface

**Fig. 4:**
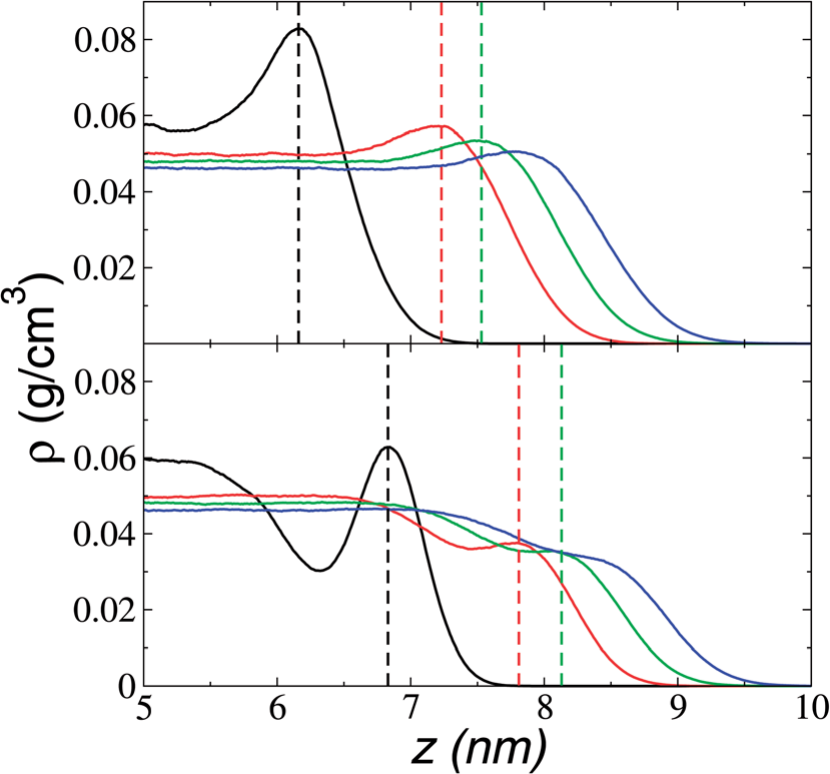
Atomic density profiles in the ionic liquid [BMIM^+^][BF_4_^−^] of carbons in the imidazole ring to which the alkyl C_4_ chains are bonded (C_R_, top) and the end carbons of the alkyl C_4_ chains (C_E_, bottom). Black, red, green, and blue lines correspond to temperatures of 373.15, 573.15, 623.15, and 673.15 K. The discontinuous and vertical lines mark the maximum value of the corresponding profiles.

The orientation of the longer alkyl chains of cations to the interfaces is estimated from the atomic density profiles of key atoms. Fig. 4 shows the profiles of the carbons in the imidazole rings to which the alkyl C_4_ chains are bonded (C_R_) and the end carbons of the alkyl C_4_ chains (C_E_). The profiles show large adsorption peaks at 373.15 K. The separation between the maximum values of the profiles is fairly constant (≈6 Å), which shows that most of the cations have their alkyl C_4_ chains pointing out of the liquid–vacuum interfaces, almost parallel to the normal direction of the interface. Paralleling the behavior of the adsorption peaks of the anions, the atomic peaks reduce their magnitude and vanish for the C_E_ profiles at the temperatures of the corresponding experimental limit of stability (673.15 K). The adsorption peaks are larger (for C_R_) and smaller (for C_E_) than the bulk liquid average values, exhibiting the low mobility of the C_R_ atoms in the normal direction and probably indicating that the chains are tilting at angles between parallel and perpendicular to the interface, with the C_R_ atoms at the base of the tilting chain. The tilting chains are free to rotate in the plane normal to the interface.

### Vaporization

**Fig. 5:**
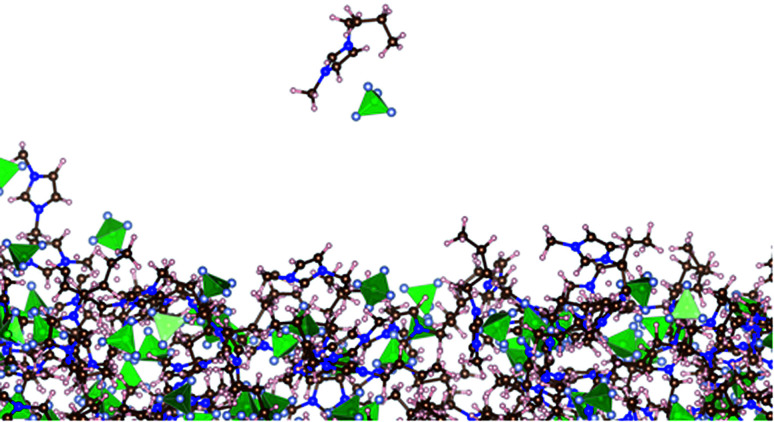
Snapshot of a vaporized neutral ion pair near the liquid layer of [BMIM^+^][BF_4_^−^] under vacuum–liquid equilibrium at 773.15 K. The brown, pink, blue, and gray circles represent carbon, hydrogen, nitrogen, and fluorine atoms, respectively.^60^ The green tetrahedrals represent boron atoms.

Very small amounts of the ionic liquid spontaneously vaporize only at the largest temperature studied (773.15 K). Fig. 5 shows snapshots of the vaporization of a neutral pair of ions after 5 ns of simulation (following equilibration). It was a rare event, which reoccurred after 11 ns, and at this time, the first vaporized pair had already condensed into the liquid due to the periodic conditions of the simulation cell. The first vaporized pair contained enough kinetic energy to permanently evaporate under the full vacuum conditions studied (without periodic boundary conditions).

### Folding/unfolding of cations

**Fig. 6:**
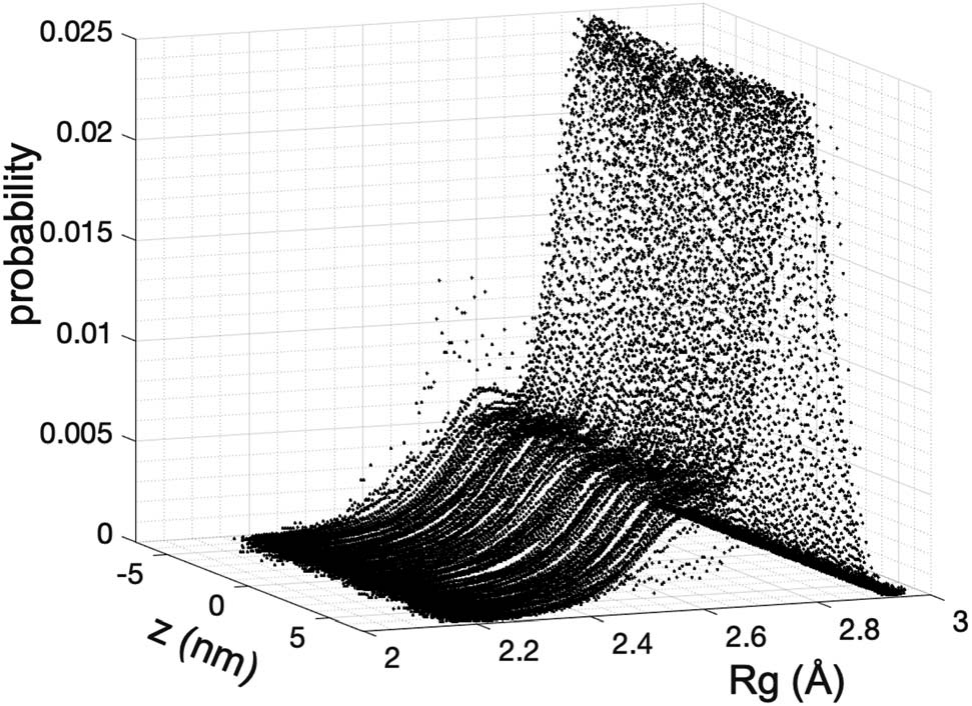
Uniform bimodal distributions of the radius of gyration (*R*_g_) of [BMIM^+^] cations in the [BMIM^+^][BF_4_^−^] ionic liquid as a function of position in the normal direction to the vacuum–liquid interface at 298.15 K. Interfaces are located at ≈−6 and +6 nm.

**Fig. 7:**
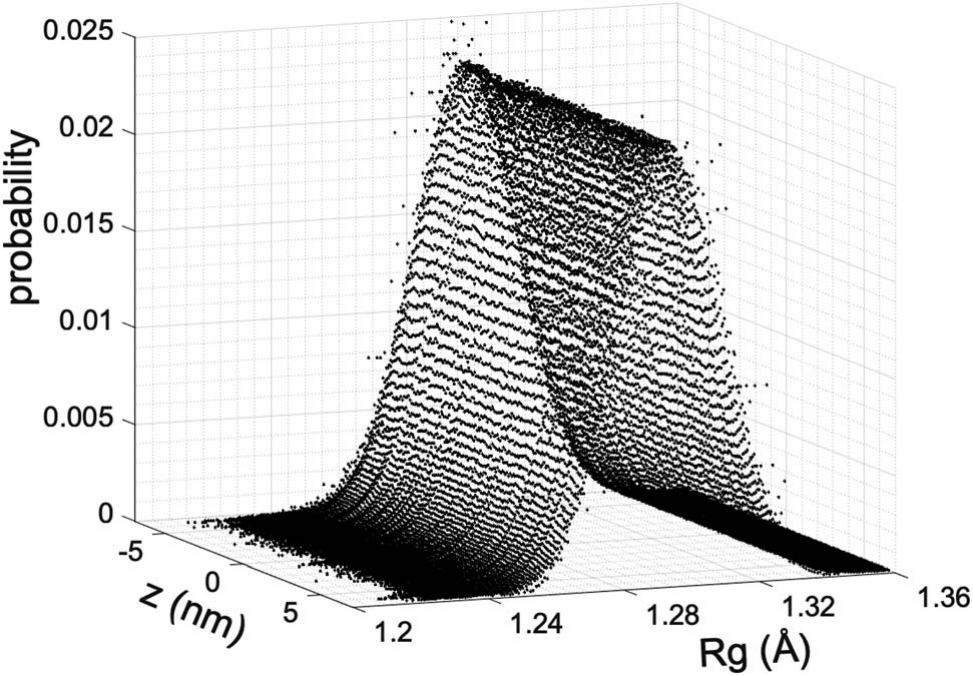
Uniform normal distributions of the radius of gyration (*R*_g_) of [BF_4_^−^] anions in the [BMIM^+^][BF_4_^−^] ionic liquid as a function of position in the normal direction to the vacuum/liquid interface at 298.15 K. Interfaces are located at ≈−6 and +6 nm.

**Fig. 8:**
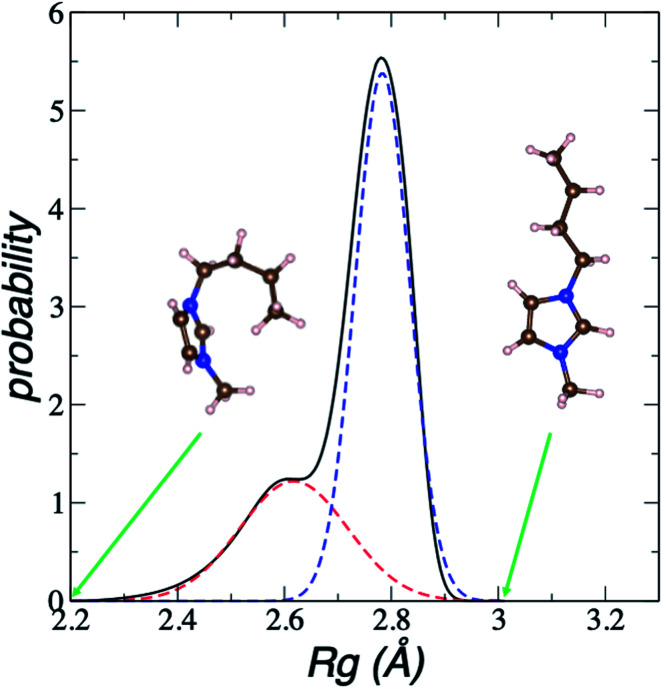
Average bimodal distribution of the radius of gyration (*R*_g_) of [BMIM^+^] cations in [BMIM^+^][BF_4_^−^] ionic liquid in vacuum–liquid equilibrium at 298.15 K, and fit to two normal distributions. The black line represents the total bimodal distribution. The red and blue dashed lines represent the best fit to a bimodal normal distribution. The snapshots illustrate conformations of the [BMIM^+^] cation at the indicated radius of gyration values. The pink, blue, and brown spheres represent hydrogen, nitrogen, and carbon atoms, respectively.^60^

We estimate the folding/unfolding distribution as a function of the temperature of the system by determining the size of the cations using the profiles of *R*_g_, which were calculated as a function of position in the normal direction to the vacuum–liquid interface, plotted for the [BMIM^+^] cations at 298.15 K in Fig. 6. *R*_g_ quantifies the size of a molecule or ion. These profiles show a bimodal distribution along the liquid layer, with variations in the outermost slabs of the profiles located at the interfaces. The *R*_g_ profiles for the [BF_4_^−^] anions (Fig. 7) exhibit normal, uniform, and narrow distributions (*σ* = 0.015 Å), which is probably due to the size of the anions. This result shows how free and unconstrained the small anions are, behaving more like a solvent in the system, surrounding and moving around the big cation structures. We produced an averaged [BMIM^+^] *R*_g_ profile from the profiles located between −5 and +5 nm; this is shown in Fig. 8. The probability of the bimodal distribution can be modeled as the sum of two normal distributions:3
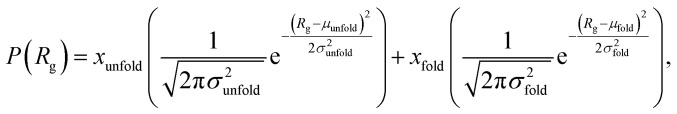
where *x*_unfold_ and *x*_fold_ represent the fractions of each conformation in each distribution, and the parameters *σ* and *μ* represent the standard deviation and mean values, respectively, of the corresponding normal distributions. Together, the two normal distributions fit the original bimodal distribution well at 298.15 K. Fig. 8 also depicts two snapshots of [BMIM^+^] cation conformations that correspond to the lowest and highest *R*_g_ values recorded during the simulations. These two normal distributions depict sets of conformations categorized as folded and unfolded (extended) conformations. Systems with more, longer chains, such as proteins^49,50^ and polymers,^51,52^ show these bimodal distributions in *R*_g_. At the mean values of the distributions, the difference in the mean *R*_g_ is small (0.17 Å), and it is difficult to determine if a specific conformation belongs to a specific distribution because the distributions overlap. At the mean values, cations are probably partially folded and partially unfolded. The distribution of folded conformations is wider, with standard deviations of ≈0.10 Å, while the unfolded conformations number half those of the folded conformations (≈0.05 Å). The parameters obtained were tested for unimodality^53^
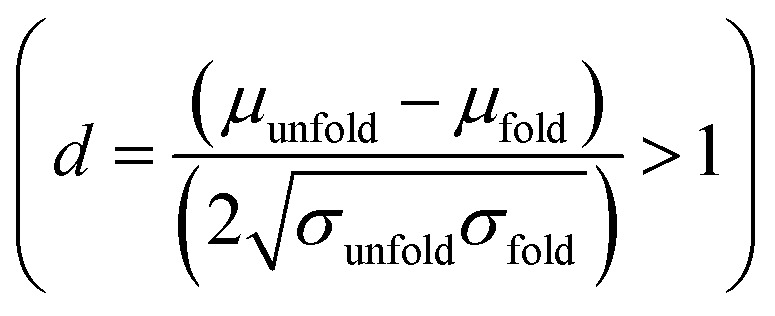
 and the result was negative; therefore, a bimodal distribution is most probable for *R*_g_.

**Fig. 9:**
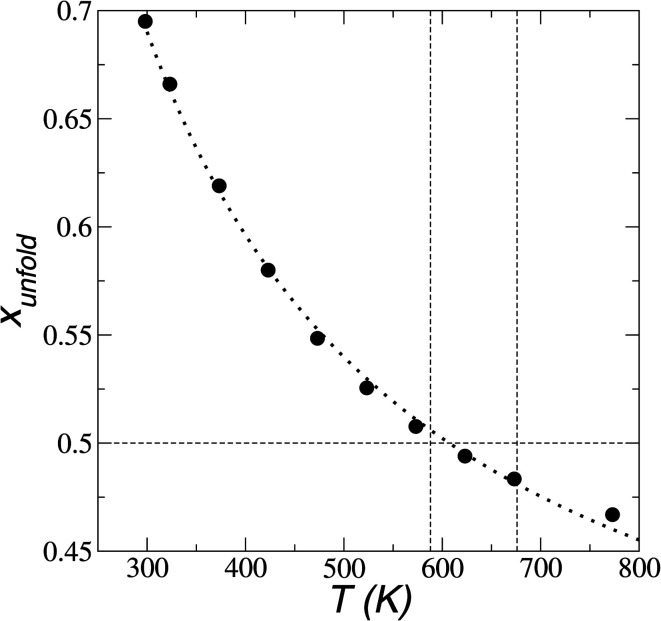
Fraction of extended conformations of [BMIM^+^] cations as a function of temperature in [BMIM^+^][BF_4_^−^] ionic liquid in vacuum–liquid equilibrium. The black circles and dotted line represent the results of this work and the best fit to an inverse function of temperature, respectively. The vertical lines represent the range of temperatures previously reported for the thermal stability of vaporization.^18–23^

In Fig. 8, the largest set (largest *R*_g_) of conformations at 298.15 K is the unfolded one. The *x*_unfold_ at 298.15 K is 0.695, which means that a significant number (≈30%) of the [BMIM^+^] cations are in folded conformations at this temperature. The *x*_unfold_ decreases as *T* increases, as shown in Fig. 9. At all studied temperatures, *R*_g_ profiles are uniform in the bulk liquid and along the interfaces, with variations in the outermost slabs. The difference between the mean values of the two normal distributions remains constant, while each distribution's standard deviation grows at a different rate as the temperature increases. The behavior of *x*_unfold_ as a function of *T* can be modeled as an inverse function in *T* (Fig. 9). Interestingly, when *x*_unfold_ crosses the equimolar barrier, the corresponding temperature is in a region that corresponds to its thermal vaporization temperature (experimental),^18–23^ which is similar to the behavior of proteins at their melting temperature, where half of the conformations of the protein are folded. The increasing share of folded conformations with rising temperature probably decreases the cohesivity of the interactions between the folded cations and the anions. Interactions between the blocking chains are facilitated, promoting interactions between cations and leaving some anions free, which ultimately reduces the cohesivity of the liquid layer. Local aggregates of alkyl chains of imidazole-based ionic liquids have been reported through experimental SAXS or WAXS studies^54^ and coarse-grained molecular dynamics simulations.^55^ The aggregates form nanoscale heterogeneities, and the length of the chain can tune their size. Cations with their larger alkyl chains folded to some degree probably facilitate the formation of local aggregates.

The dissociation of [BMIM^+^] cations becomes more important as the temperature of the system increases, which likely favors the thermal stability of the system because free imidazole cations are formed as a result of dissociation, and free imidazoles cannot be blocked (by folding chains) to interact with the anions. Ionic liquids with more rigid chains will likely be more thermally stable over a wider range of temperatures because those chains require more energy to bend.

### Pressure profiles and surface tension

**Fig. 10:**
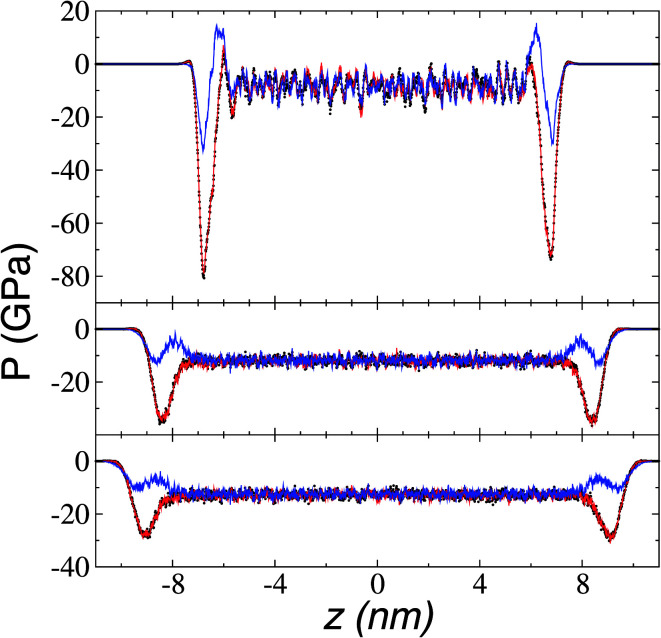
Normal (blue) and transverse (red and black) pressure profiles as a function of position in the normal direction to the vacuum–liquid interface for [BMIM^+^][BF_4_^−^] ionic liquid at 373.15 K (top), 673.15 K (middle), and 773.15 K (bottom).

We calculated the pressure profiles to investigate the interfacial forces in operation as the system reaches its thermal stability limit. The profiles in the normal (*P*_n_) and transverse (*P*_t_) directions are illustrated in Fig. 10 for temperatures lower than, at, and near the limiting temperature for thermal stability. The average bulk pressures show negative values in all directions, which are expected in fluids under total vacuum and equilibrium. At all temperatures, the *P*_t_ profiles show the characteristic negative peak related to the cohesivity of the system in this direction, the magnitude of which decreases as temperature increases. The cohesive *P*_t_ peak remaining at the thermal limit probably indicates that, after the system crosses this limiting temperature, the transformed system (probably to a superheated liquid) has cohesive interfaces that can manifest as surface energy in the transverse directions. In contrast, at temperatures below the thermal stability temperature *P*_n_ transforms from a region of two peaks with opposite signs at the interface to a region with intermediate negative values at the limiting temperature. The two-peak zone at temperatures below the thermal limit is associated with zones of different cohesivity—an outermost, external interfacial zone (negative peak), where the bulk liquid zone strongly attracts chains of cations in ionic liquids,^56^ chains in alkanes and polymers,^45^ or atoms in atomic systems.^43,44^ Interactions between the outermost cohesive zone and the bulk liquid create an intermediate crunched zone, which manifests as the positive peak. Starting at 673.15 K, the outermost external negative peak of *P*_n_ shows values lower than those of the bulk liquid, probably indicating how metastable the systems are at these temperatures. The reported slow vaporization rates at 773.15 K, with highly energetic neutral pairs, confirm that these systems are in the process of fully changing phase at these thermophysical conditions.

**Fig. 11:**
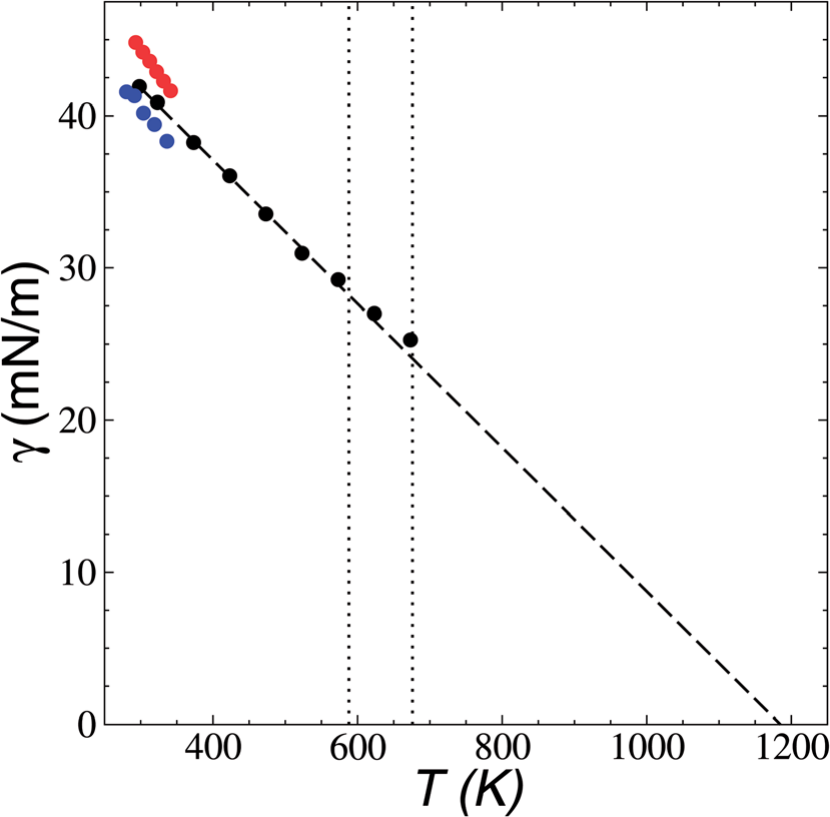
Surface tension (*γ*) as a function of temperature in [BMIM^+^][BF_4_^−^] ionic liquid in vacuum–liquid equilibrium. The black circles represent the results of this work. Red and blue circles represent experimental results.^57,58^ The dashed line represents the linear fit. The vertical lines represent the range of temperatures reported for the thermal limit of stability.^18–23^

**Fig. 12:**
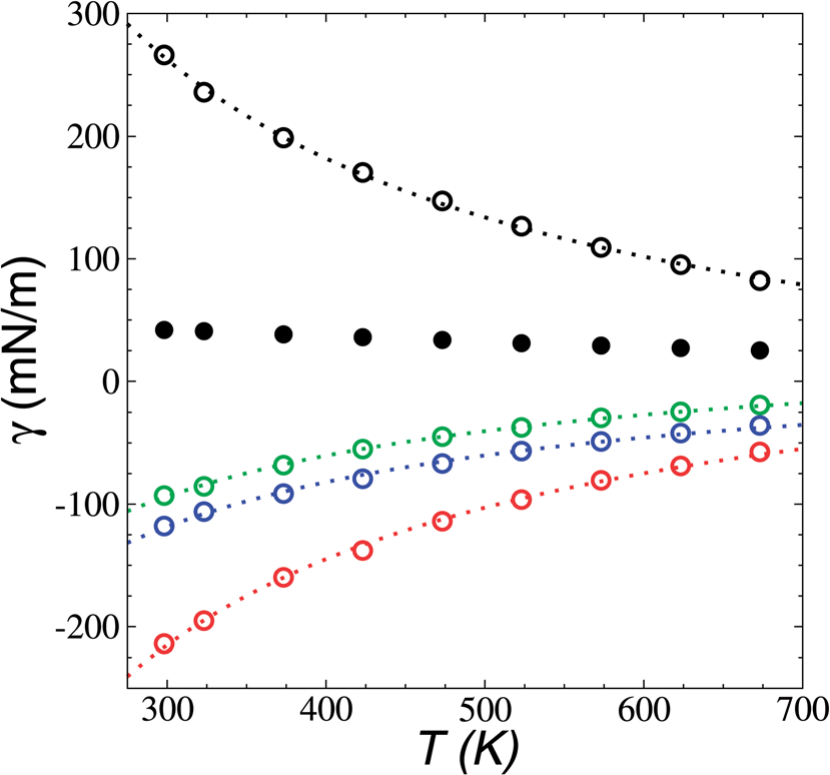
Surface tension (*γ*) contributions as a function of temperature in [BMIM^+^][BF_4_^−^] ionic liquid in vacuum–liquid equilibrium. Filled circles represent the total surface tension. Open black, red, green, and blue circles represent intermolecular (coulombic + dispersion), intramolecular (bond + angle + dihedral + improper), and bond and angle interactions, respectively. Dotted lines represent the best fits to an inverse function of the temperature.

Surface tension was calculated using its mechanical definition:4
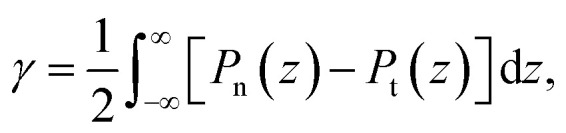


the resulting average surface tension of the liquid layers was linear for temperatures below the thermal limit of stability. The simulation data agreed with previously reported experimental results^57,58^ for *γ* at temperatures in the range of 293.15–341.45 K (Fig. 11). The experimental datasets showed a parallel (linear) behavior, with different intercepts at the axes, showing differences of up to 3.7 mN m^−1^ (≈8%) at 293.15 K. The simulation results are located between the two experimental datasets. Extrapolations at temperatures where *γ* vanishes predicts a “critical temperature” of 1185 K, which has been predicted between 1158 and 1240 K, depending on the expression used to fit the experimental data.^59^ The contributions to *γ* (Fig. 12) showed the expected positive contributions from intermolecular interactions and negative contributions from the intramolecular interactions, which reached a plateau at the limit of thermal stability but they do not fully cancel, which is probably a manifestation of the metastable state reached, and probably due to the short time length of the simulation. The main intramolecular interactions are the bond and angle interactions, and the negative values likely indicate how stressed the ion structures are under these conditions.^45^

## Conclusions

The results reported in this work show that the cation conformations of the bulk liquid and at the vacuum–liquid interface show complex behavior. There is a dual distribution (folded/unfolded) of cation conformations in the [BMIM^+^][BF_4_^−^] ionic liquid at temperatures as low as room temperature. The calculation of the profile of the radius of gyration revealed that 30% of the cations are in folded conformations in the bulk liquid at 298.15 K. In contrast to the behavior of the thermal denaturation of proteins, the concentration of folding conformations increases as the temperature rises. When an equimolar concentration is reached, the system reaches the transition temperature (vaporization temperature). At temperatures at and above 673.15 K, there are indications that the system is metastable and becomes mechanically unstable in the normal direction and while remaining stable in the transverse directions. Another indication of metastability is the observed vaporization of highly energetic (kinetic) neutral pairs at 773.15 K at a very slow rate of vaporization without forming larger aggregates in the vapor phase. At the interfaces, another phenomena indicate that the system is in the thermal limit of stability, *i.e.*, separation between the two distinctive layers of cations and anions disappears, adsorption peaks at the interfaces present in the total, ionic and atomic density profiles vanish in the region of thermal stability, and the number of cations showing the preferred orientation also disappears at the thermal limit.

Future work should clarify the impact of the folding/unfolding phenomena in ionic liquids on properties such as free energy, enthalpy, and entropy. We will also study the effect of hydrophobic or hydrophilic impurities on the interfacial behavior of the hydrophobic alkyl chains and the vaporization process of neutral pairs of ions, and the effect of larger thermal and mechanical fluctuations in larger systems.

## Conflicts of interest

The authors declare no conflicts of interest.

## Supplementary Material
